# Influence of Pore-Size Distribution on the Resistance of Clay Brick to Freeze–Thaw Cycles

**DOI:** 10.3390/ma13102364

**Published:** 2020-05-21

**Authors:** Ivanka Netinger Grubeša, Martina Vračević, Jonjaua Ranogajec, Snežana Vučetić

**Affiliations:** 1 Faculty of Civil Engineering and Architecture Osijek, Josip Juraj Strossmayer University of Osijek, Vladimira Preloga 3, 31000 Osijek, Croatia; 2 Laboratory for Materials Testing, Civil Engineering Institute of Croatia, Drinska 18, 31000 Osijek, Croatia; martina.vracevic@igh.hr; 3 Faculty of Technology, University of Novi Sad, bul cara Lazara 1, 21000 Novi Sad, Serbia; janjar@uns.ac.rs (J.R.); snezanap@uns.ac.rs (S.V.)

**Keywords:** clay bricks, resistance to freeze–thaw cycles, raw materials, shaping procedure, firing regime, pore-size distribution, compressive strength, Maage factor

## Abstract

This study examines the influence of raw material characteristics, methods of shaping and of parameters of firing process of clay bricks, on pore-size distribution and on resistance to freeze–thaw cycles (with particular emphasis on the retention time of the specimens at the maximum achieved temperature). Pore-size distribution was measured by mercury-intrusion porosimetry, while the resistance to freeze–thaw cycles was assessed by exposing the bricks to freeze–thaw cycles (HRN B.D8.011 standard) monitoring the appearance of surface changes, decrease of compressive strength as well as the Maage factor. A correlation was set up between the Maage factor and the ratio of the compressive strength before and after freezing as a quantitative indicator of bricks resistance to frost. By using this correlation for all the examined bricks, regardless of their raw material and shaping procedure, a low coefficient of correlation (R^2^ = 0.26) was obtained. When processed separately, machine-made bricks had a significantly higher correlation coefficient value (R^2^ = 0.60) than the hand-made bricks (R^2^ = 0.28).

## 1. Introduction

One of the main requirements of brick wall elements is their durability. Salt crystallization and cycles of water freezing–thawing are considered to be among the main factors of brick degradation [1,2,3]. Porous building materials such as brick wall elements always contain a certain amount of moisture in their structure, which directly influences the material properties such as strength, shrinking/expansion properties, vapor permeability and resistance to external conditions. According to the European regulations, the resistance of bricks to freeze–thaw cycles is assessed through CEN/TS 772-22 standard [4]. However, in addition to the aforementioned direct method of testing the resistance of bricks to freeze–thaw cycles, the literature mentions some indirect procedures/methods and limits critical values per procedure/method to grade the resistance of bricks in freeze–thaw cycles. A highly acknowledged indirect procedure for predicting the resistance of bricks to freeze–thaw cycles is the well-known Maage coefficient [5,6,7,8,9,10]. The Maage coefficient is based on experimental results and presents a statistical model with two main variables: the total volume of pores (PV), and the share of pores of a certain diameter, i.e., pores larger than 3 µm (P3). According to Maage, pores larger than 3 μm in diameter have a beneficial effect on frost resistance of bricks. Other authors claim that it is precisely the large pores that are responsible for the good resistance of clay bricks to freeze–thaw cycles [1,3,11,12,13,14]. Furthermore, small pores are not considered to significantly affect the resistance of brick to freeze–thaw cycles, while those of medium size are considered to be critical pores [1,3]. According to Elert et al. [11], small pores are defined as ones whose diameter is <0.2 μm, medium-sized pores are of diameter 0.2–2 μm while large pores are of diameter >2 μm. Pores with diameters <0.25 μm are considered to be small pores and those with diameter >1.4 μm as large pores according to Ravaglioli [15], while Koroth [1] and Kung [16] consider pores in the range 0.1–1.0 μm to be critical (medium-sized pore). Culturone et al. [12] divided pores in two groups; pores with radius <1 μm (small pores or micropores) and pores with radius >1 μm (large pores or macropores) considering the small pores harmful. The presence of small pores negatively affects the quality of bricks, since their capacity to absorb and retain water increases [12]. Stryszewska and Kanka [14] divided pores into as many as five groups (<0.1 μm, 0.1–1 μm, 1–3 μm, 3–10 μm and >10 μm). They set up a correlation between the particular form of frost damage and the prevailing pore group. The final conclusion in their research is that bricks without any signs of damage are clearly characterized by the prevalence of pores with diameters in the range of 3–10 μm and that the pores with diameter <0.1 μm do not affect the resistance of brick to freeze–thaw cycles.

Pore-size distribution in a brick unit is influenced by the characteristics of raw materials mixture, shaping procedure (hand-made or machine-made bricks) and thermal treatment. Elert et al. [11] have studied the frost resistance of hand-made bricks shaped from two different raw materials (calcareous and non-calcareous clay) and fired at 700, 800, 900, 1000 and 1100 °C each. The conclusion of their research was that the durability of non-calcareous bricks fired at 1000 °C or above is generally superior. These bricks display lower total porosity and a higher degree of vitrification than the corresponding calcareous specimens. For non-calcareous clays, a firing temperature of 1000 °C is high enough to produce durable bricks, while a temperature of 1100 °C is necessary in the case of calcareous clays. Elert et al. [11] observed that brick specimens with high porosity and a high percentage of pores with a diameter of <2 μm underwent significant damage during freeze–thaw cycles. The authors in [12,17,18] also concluded that the clay with a lower CaCO_3_ content would ensure more exceptional durability as the final products. For raw materials with slightly higher carbonate content, it is necessary to increase the firing temperature in order to reduce the amount of medium-sized (harmful) pores, which are formed by CO_2_-separation from the raw material [5,18].

The authors in [19,20,21] claim that hand-made bricks contain a higher content of large pores than machine-made bricks, which renders hand-made bricks more resistant to freeze–thaw cycles. On the other hand, the research presented in [22] clearly shows that machine-made brick contains significant content of large pores. It should be noted here that the hand-made and machine-made bricks explored in [22] were not made from the same raw material. Machine and hand-made bricks produced from the same raw material were studied in [23] and it is observed that hand-made bricks achieved a higher content of large pores than machine-made bricks fired at the same temperature. The basic difference between machine and hand-made production of bricks, based on the same raw material, lies in their textural properties. Elert et al. [11] and Netinger et al. [23] studied the influence of firing temperature on the pore structure of clay bricks and concluded that the total porosity of brick specimens decreases and pore-size distribution changes towards larger pore sizes as the firing temperatures rise. The authors in [24] came to the same conclusion regarding the pore structures of clay roofing tiles. The retention at the maximum firing temperature is also an important parameter considering the structural characteristics of the final product. A more extended retention period has evidently a positive influence on the final properties of the product [25].

In order to assess clay product’s resistance to freeze–thaw cycles, researchers have been studying the changes of the following properties during freeze–thaw cycles: surface appearance of the specimens [14,23,24,26], flexural strength and toughness [24], compressive strength and dynamic modulus of elasticity [27], propagation speed of ultrasonic waves through specimens [12,27], weight of specimens [11,26,27,28] and the structure of pores [24]. During freeze–thaw cycles, the surface of brick specimens becomes damaged, compressive and the flexural strength (as well as dynamic modulus of elasticity, toughness and weight) is decreased, and so is the propagation speed of ultrasonic waves through the specimens. With each freeze–thaw cycle, new micropores and cracks appear.

As the literature shows, the influence of pore-size distribution on brick resistance to freeze–thaw cycles has been an interesting topic for many years. However, the literature fails to cover all the factors that simultaneously influence the frost resistance of a brick, a process that is not simple and easy to investigate. The authors of this paper study the simultaneous influence of the following factors: the characteristics of raw material, shaping method and influence of firing regime (with particular emphasis on the retention time) on frost resistance of the bricks exposed to freeze–thaw cycles, by monitoring the changes in surface appearance and the changes of compressive strength as well as the Maage factor. A correlation was set up between the Maage factor and the ratio of compressive strength before and after freezing as a quantitative indicator of brick frost-resistance.

## 2. Materials and Methods

### 2.1. Characterization of Raw Materials

Two mineralogically different raw materials (S1 and S2), from Kukljaš clay pit in the region of Osijek, Slavonia, Croatia, were used for the manufacturing of the bricks. Chemical quantitative analysis of these raw materials was done by using X-ray fluorescence spectroscopy—XRF analysis. The equipment, ARTAX 200 µ-XRF spectrometer (BRUKER Nano, Karlsruhe, Germany), was provided with a Rh cathode ray tube and an integrated camera. The spectra were obtained under the following experimental conditions: voltage of 25 kV, current intensity of 1500 µA, scanning time in the duration of 100 s in the helium atmosphere condition. The obtained spectra were analyzed by using the integrated ARTAX SPECTRA 7 software developed by BRUKER. The described portable machine works in a contactless mode at a distance of 2 cm where the angle of emitted X-rays is 45° in relation to the test area. The measurements of the elemental analysis were done on 10 different points with the energy of the electron beam of 15 keV (amplification of 200 times). The obtained results are given in Table 1, where the values of the peaks are labeled with Net, while the element concentrations with Conc.

The chemical composition of the raw materials, by using scanning electron microscopy (FE_SEM) and energy-dispersive X-ray spectroscopy (EDS), are expressed in the form of oxide, Table 2.

**Figure 1:**
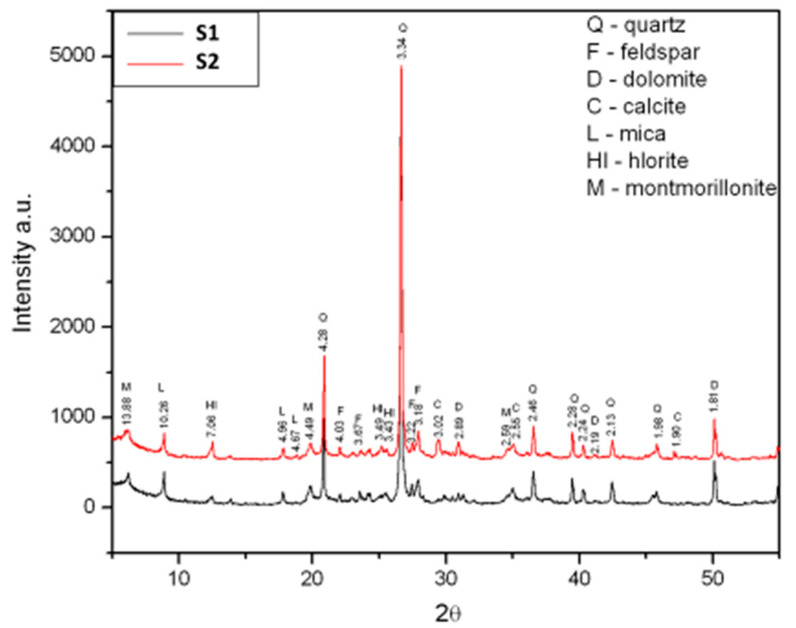
Comparative analyses of two raw materials X-ray structural analysis (XRD) diagrams.

The mineralogical composition, Figure 1, of the raw materials was determined by using X-ray structural analysis (XRD) while the content of combustible and organic substances was determined according to the HRN *U*.B1.024 standard [29]. The content of carbonates was assessed based on the HRN *U*.B1.026 standard [30], Table 3.

**Figure 2:**
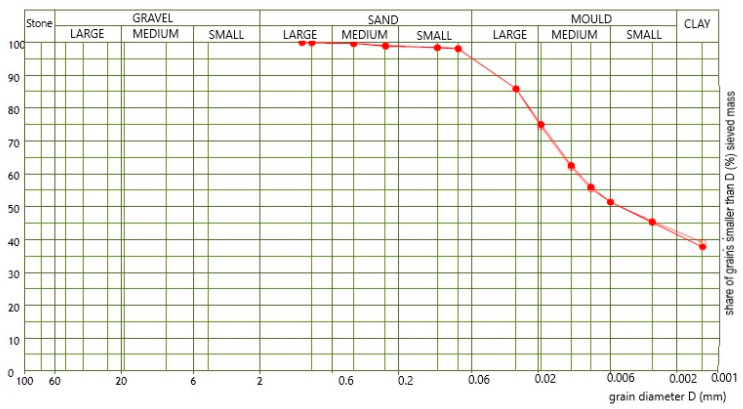
Particle-size distribution of two raw materials.

Distribution of particles of the raw materials was determined according to the ASTM D 422–63 standard [31], and the results of the testing are shown in Figure 2 and Table 4. The dispersion of the sample for the hydrometer method was done in an electric mixer; the duration of mixing was 1 min.

The results of the mineralogical analysis shown in Figure 1 point to the presence of the following minerals: quartz (Q), feldspar (F), dolomite (D), calcite (C), mica (L), chlorite (Cl) and montmorillonite (M) in the case of both raw materials. The results of the chemical and mineralogical analysis show that both raw materials have a very similar composition, with a small difference in the amount of CaO: 1.56 mass% in the raw material S1 and 3.24 mass% in the raw material S2). Clays with the CaO content under 6 mass% [32] are considered to be low-carbonate materials. Therefore, both raw materials can be characterized as low-carbonate—with a slight difference in the amount of the CaO content. The granulometric curves for both raw materials (Figure 2) are very similar, with a slight difference in the number of small particles, i.e., clay. The test results of the mineralogical structure of the raw materials, together with their granulometric compositions, show that the raw materials are sandy raw materials, with the presence of a small quantity of carbonates, rich in clay minerals.

**Figure 3:**
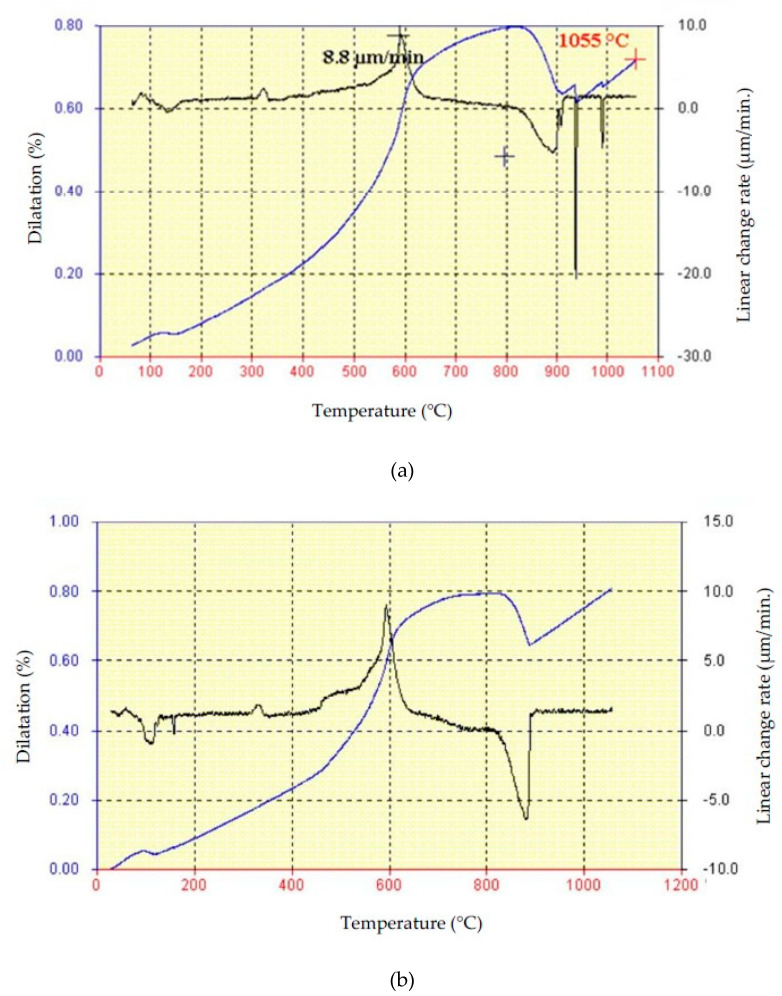
Results of the dilatometric analysis of raw materials (**a**) S1 and (**b**) S2.

Furthermore, the samples of raw materials S1 and S2, prepared according to ASTM C824-91 [33], were tested for their dilatometric properties. The samples were heated at a rate of 3 °C/min. The results of the testing are shown in Figure 3a,b. The blue curve (integral) shows the relative change of the sample with the temperature expressed in percentages, while the black curve represents the differential dilatometric curve, i.e., linear change expressed in µm/min.

The results of the dilatometric analysis, with the previous knowledge of the mineralogical composition of the raw materials, point to some characteristic effects: extracting moisture adsorption, dehydroxylation of clay minerals, ignition of organic substances, decarbonization as well as the process of sintering/vitrification process. The first change (shrinking) on the dilatometric curve, in the case of both samples, occurs at 100 °C, which is the consequence of the loss of the adsorption moisture in the analyzed raw materials [5,32]. The combustion (oxidation) of the organic substances is visible at 300 °C in the case of both raw materials, which is accompanied by a linear expansion of the samples. By further increase of temperature, the linear expansion can be detected until 550–600 °C, where the differential dilatometric curve shows a corresponding distinctive effect. This effect occurs because of the transformation β into α quartz and the dehydroxylation of clay minerals. The expansion of the samples is continuous at the temperature between 700 and 800 °C. Namely, at temperatures of about 700 °C the process of decarbonization begins, first of calcite and then of dolomite, where a certain amount of CO_2_ is created contributing to the expansion of the samples [34]. The maximum expansion of 0.74% was identified for the raw material S1, and a slightly higher value, 0.80%, for the sample S2. A sudden shrinking of the samples, due to the completion of montmorillonite structure decomposition, is noticed at temperatures above 800 °C. The process of vitrification starts up at around 900 °C [32,34]. According to Elert et al. [11] a more significant vitrification happens for raw materials with a lower carbonate content, as is the case of the raw material S1 where this phenomenon happens in two steps, at 940 °C and at 990 °C.

### 2.2. Brick Manufacturing

**Figure 4:**
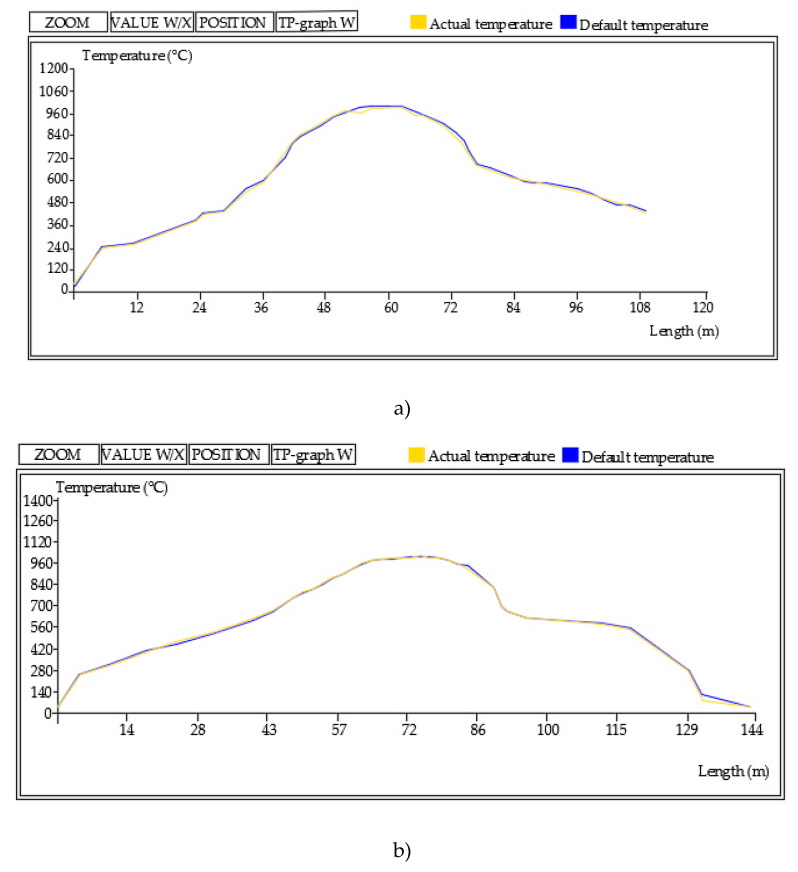
Firing regime of the bricks (**a**) at 1030 °C and (**b**) at 1060 °C.

From each of the two raw materials (S1, S2), two sets of bricks were made: one by hand (hand-made bricks) and the other on an industrial line (machine-made bricks). The hand-made bricks were made on the manufacturing line of the company “Old art décor“ d.o.o., Josipovac, Croatia, while machine-made bricks, measuring 250/120/65 cm, were produced on an industrial line at “OPEKA” d.d. brick company Osijek, Osijek, Croatia. There were 100 specimens of bricks made by hand and 100 industrial bricks from each raw material (S1, S2). All brick specimens were dried for 45 days. They were placed on a flat ground covered with sand to prevent the sudden loss of moisture. After drying, the firing process followed in an industrial kiln (Hans Lingl Anlagenbau und Verfahrenstechnik GmbH & Co. KG kiln from 2003, Lingl, Krumbach, Germany). The used firing regimes are shown in Figure 4.

The maximum of the firing temperatures (1030/1060 °C) was determined based on the characteristics of the raw materials and of the results of Elert et all [11]. Namely, these authors studied the frost resistance of non-calcareous and calcareous clay bricks fired at 700, 800, 900, 1000 and 1100 °C analyzing the pore structure of each brick group. However, there was a large temperature gap between the two last temperatures in the case of their investigation. As this temperature interval is significant for vitrification and formation of new crystal forms, which have an enormous influence on frost resistance characteristics, the authors decided to choose 1030 °C as the maximum firing temperature for the raw materials with lower carbonate content (S1), while the authors chose 1060 °C for S2 raw material. It was assessed that this temperature would be high enough to develop a satisfied quantity of pores with a diameter >3 µm and reduce the volume share of the medium-sized pores. Within each of the four batches of unfired bricks (2 types of raw material × 2 types of manufacture), the retention time of bricks at the highest achieved temperature was different, 90 min and 30 min, respectively.

### 2.3. Brick Testing

#### 2.3.1. Pore-Size Distribution

Hg porosimetry [35] of unfired and fired bricks was conducted by using an AutoPore IV 9500 device, model 9500 (Micromeritics, Norcross, GA, USA), with the possibility of achieving the pressure up to 33,000 psi (228 N/mm^2^), pore radius from 360 to 0.001 µm. The distribution of pores in four batches of unfired bricks (2 raw materials × 2 types of brick manufacture) and the total volume of pores (PV) in the range of pore radius from 360 to 0.001 µm was determined with the above-mentioned porosimeter. The same parameters were determined for eight batches of the fired bricks (2 types of raw material × 2 types of brick manufacture × 2 different retention times in the kiln at the highest achieved temperature).

#### 2.3.2. Testing the Resistance of Fired Bricks to Freeze–Thaw Cycles

The resistance of bricks to freeze–thaw cycles is determined according to HRN B.D8.011 standard [36]. The regime described in this standard is corresponding to the method described in the former Finnish standard [37] which is more rigorous than the CEN/TS 772-22 standard [38] and which causes measurable structure changes in the brick body. The authors in their previous study [39] dealt with this standard considering that the CEN/TS 772-22 standard was not strict enough for the climate with a high level of rainfall combined with numerous freeze–thaw cycles during winter. In addition, the HRN B.D8.011 standard requires a smaller number of testing specimens (brick units) compared to the CEN/TS 772-22 standard [4]. Considering the chosen standard, the specimens were saturated with water and exposed to temperatures −20 ± 2 °C for four hours in a climate chamber. Subsequently, the specimens were submerged in water at a temperature of +15 to 20 °C, also for four hours. This cycle was repeated 25 times and the specimens were examined after every cycle. The brick is considered durable to freeze–thaw cycles if after 25 cycles of freezing and then defrosting in water, there are no signs of damage in any of the examined specimens. However, since this method gives a qualitative, and not a quantitative assessment, for an additional assessment of the brick resistance to freeze–thaw cycles the changes in compressive strength (expressed as the ratio of compressive strength of the bricks before and after freezing) were studied. For this purpose, compressive strength values of the bricks were determined according to EN 772-1 [40]. Average values of the compressive strength before and after freeze–thaw were considered for a future investigation.

## 3. Results

**Figure 5:**
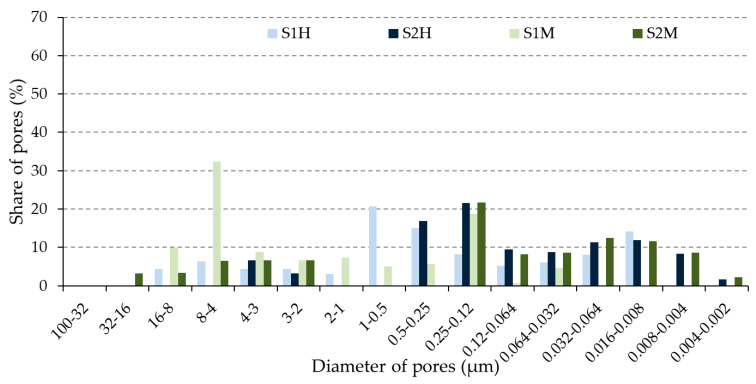
Pore-size distribution in the unfired bricks.

The distribution of pores of the unfired bricks for each specified size, determined by Hg porosimetry, is shown in Figure 5. Based on the reference literature, pores with a diameter of less than 0.1 µm are considered to be small, while the ones with the diameter over 1.0 µm are considered to be large [1,16]. Maage [5,6,7,8,9,10] in his equation considers pores to be large only if their diameter is over 3.0 µm. Based on this fact, the authors decided to use the Maage methodology and his equation will be considered for further analysis. This approach was used for both the unfired and fired bricks. In line with this, medium-sized pores are considered to be those with the diameter in the range of 0.1–3.0 µm. The used specimen labels are given in Table 5.

**Figure 6:**
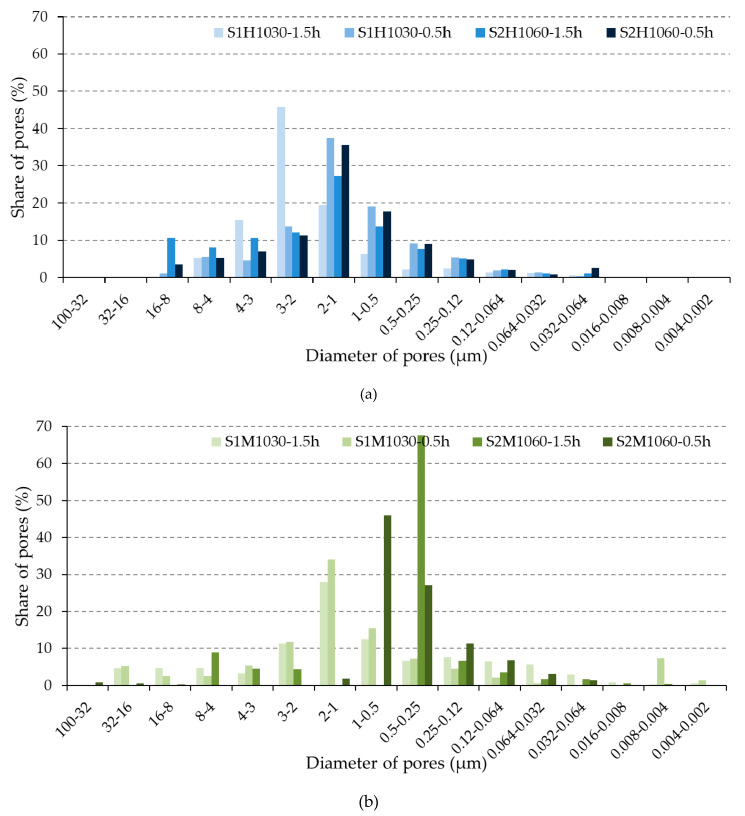
Pore-size distribution in the (**a**) hand-made and (**b**) machine-made fired bricks.

**Figure 7:**
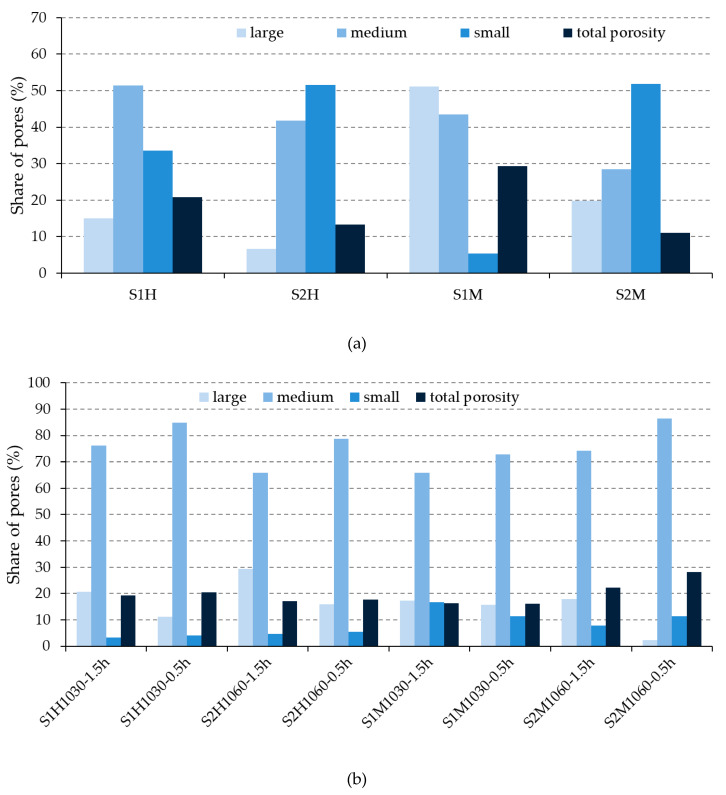
Pore-size distribution and total porosity of the (**a**) unfired bricks and (**b**) fired bricks.

The pore-size distribution of the unfired and fired brick specimens is given in Figure 5 and Figure 6. Furthermore, Figure 7 shows pores of each size, according to Maage methodology categorized into groups of large–medium–small, in the unfired and fired bricks. This Figure was designed based on the previous Figure 6, that describes the pores’ dimensions into the interval 100–0.002 μm (Hg-porosimetry).

**Figure 8:**
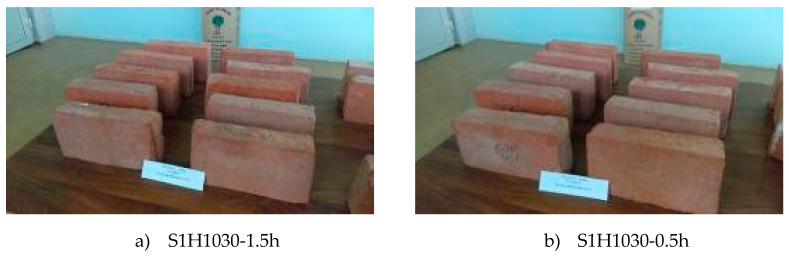

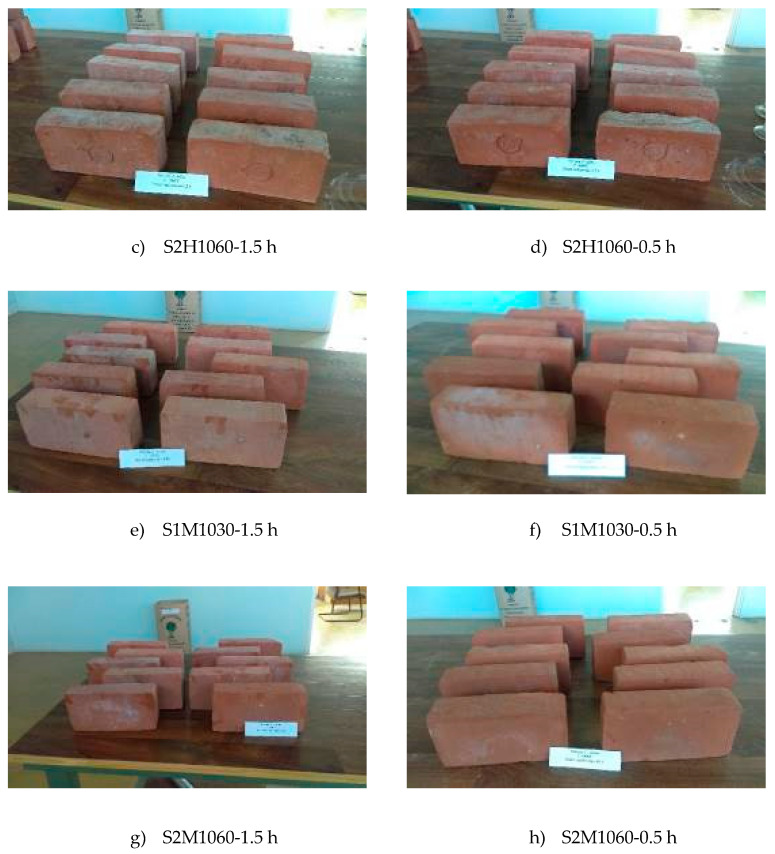
Bricks after freeze–thaw cycles.

The appearance of the bricks after the exposure to freeze–thaw cycles are shown in Figure 8.

**Figure 9:**
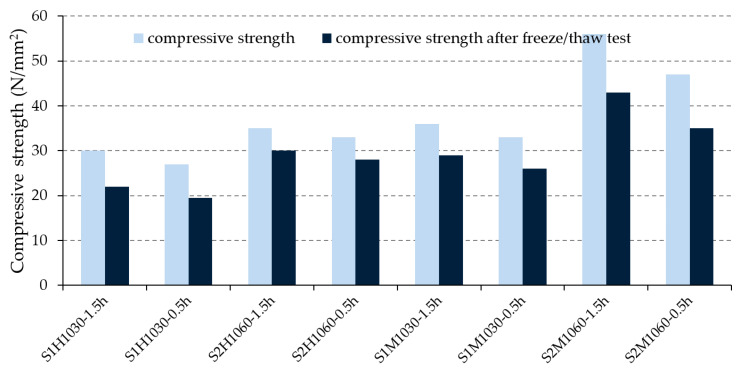
Compressive strengths of the bricks before and after freeze–thaw cycles.

Values of compressive strengths (average of ten measurements) of the bricks before and after the exposure to freeze–thaw cycles are presented in Figure 9 while these values in comparison with the ratio of compressive strength before and after the cycles are show in Table 6.

## 4. Discussion

Figure 9 shows that the bricks made from S2 raw material achieved higher compressive strength than the ones made from S1 raw material fired at the maximum reached temperature (1060/1030 °C). As the microstructure of S1 bricks was obtained based on the vitrification process [34,41], evidently, the content of glass phase decreased the mechanical value of the bricks. Considering the retention time of bricks at the highest temperatures, the obtained results showed that this parameter has a positive influence on the value of compressive strengths value. This fact is the consequence of the formed microstructure with a higher content of larger pores and a lower content of dangerous medium and small pores, Figure 7.

Based on the appearance of the bricks after their exposure to freeze–thaw cycles (Figure 8), it was concluded that none of the brick groups had any damage caused by freezing/thawing procedure and all were assessed as resistant. However, the ratio of compressive strength before and after freeze–thaw test (Table 6) shows that some bricks have undergone more pronounced inside changes. According to the literature cited in the Introduction [11,12,17,18], a clay material with a lower CaCO_3_ content should ensure better durability of the final products than the one with a higher content of CaCO_3_. Based on the results, only the machine-made bricks confirmed this conclusion. As shown in Figure 7b), the machine-made bricks produced from S2 raw material developed a similar share of large pores (except the group of bricks S2M1060-0.5h) as those made from S1 raw material, but a larger share of medium-sized pores (harmful pores), which proves a better freeze–thaw resistance of the bricks made of S1 clay.

The opposite was true for the hand-made bricks: the ratio of medium pores was lower for bricks made of clay with a higher content of CaCO_3_ (S2) (e.g., S1H1030-1.5h vs. S2H1060-1.5h; S1H1030-0.5h vs. S2H1060-0.5h), but the share of large pores was higher. Considering that the trends in the pore ratios were different for hand and machine-made bricks, it was not possible to conclude whether the raw material unambiguously affected the durability values or if it was due to the shaping procedure.

Analyzing the results from Figure 7a, it is evident that the raw materials have a more significant effect on the total share of pores than on their distribution. In fact, in the case of the fired bricks made from S1 raw material, the total porosity decreased in comparison with the unfired bricks, while in the case of the fired bricks made from S2 raw material, the total porosity increased in comparison with the unfired ones. This is the reason why machine shaping bricks made from S2 raw material, even though they achieved better compressive strengths in comparison with the ones made from S1, did not reach a satisfactory frost resistance.

Comparing the ratio of compressive strengths (Table 6) of the bricks made from the same raw material and with the same retention time at the maximum reached temperature, but shaped differently (hand-made/machine-made) (S1H1030-1.5h vs. S1M1030-1.5h; S2H1060-1.5h vs. S2M1060-1.5h), it could not be concluded that shaping procedure directly affect their resistance to freeze–thaw cycles, which is contrary to the conclusions given in [19,20,21]. Evidently, the shaping procedure influenced the structure of pores in the case of the unfired bricks (Figure 7a), but the microstructure changes, after the firing process, defined their final resistance.

Reviewing the ratio of compressive strengths before and after the freeze–thaw cycles, it could be firmly concluded that the bricks with a longer retention time at the maximum temperature (1.5 h), generate a better resistance to freeze–thaw cycles than bricks with a shorter retention time (0.5 h): the ratio of compressive strengths was higher in the case of S1H1030-1.5h than in S1H1030-0.5h, again higher in the case of S1M1030-1.5h than in S1M1030-0.5h. The explanation could be found in the change of microstructure but also of the pore structure, Figure 7b. These results point out that the bricks retained longer at the maximum temperature (1.5 h), regularly report a greater share of large pores and a smaller share of medium pores than the bricks made from the same raw material, using the same type of manufacture (hand-made or machine-made), but fired at the same temperature for a shorter amount of time (e.g., S1H1030-1.5 h vs. S1H1030-0.5 h; S1M1030-1.5 h vs. S1M1030-0.5 h). This result is compatible with the reference literature [5,6,7,8,9,10].

Taking into account the share of large pores (P3), Figure 7b and the total volume of pores (PV), the Maage factor, F_C_ = 3.2 × PV + 2.4 × P3, for the analyzed group of bricks was calculated, Table 6.

In addition to the Maage factor (F_C_), Table 6, shows again the assessment of brick resistance to freeze–thaw cycles according to HRN B.D8.011 [36]. This presentation summarizes the obtained results of the used methods in order to assess the brick resistance to freeze–thaw cycles.

**Figure 10:**
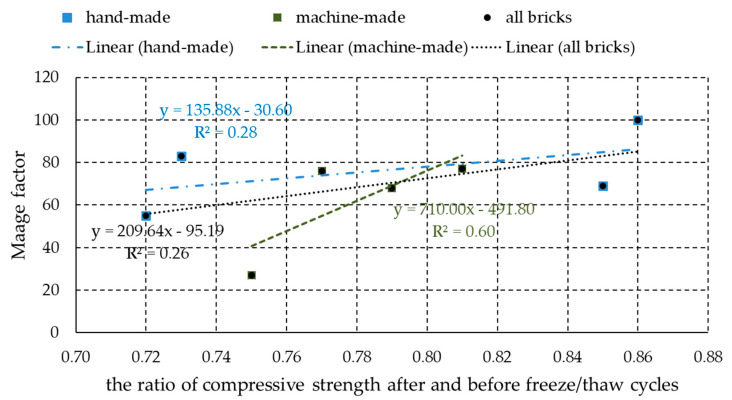
Ratio of compressive strengths before and after freeze–thaw cycles vs. the Maage factor.

It is evident (Table 6) that the bricks, which are assessed to be resistant to freeze–thaw cycles according to HRN B.D8.011 [36], have the ratio of compressive strengths over 0.72 and the Maage factor in range 27 to 100. Based on the reference literature [5,6,7,8,9,10], only the bricks with the Maage factor higher than 70 are considered resistant to freeze–thaw cycles. However, in the research, even the bricks with a smaller Maage factor (68:27) proved to be resistant. While trying to make a relation between the ratio of compressive strengths and the Maage factor (Figure 10), a low coefficient of correlation of R^2^ = 0.26 for the equation y = 209.64x − 95.19 (y is the Maage factor and x is the ratio of compressive strengths) was obtained in the case that all bricks were analyzed together regardless of the method of brick forming. However, if the results are processed separately for hand-made and machine-made bricks (Figure 10), it is evident that machine-made bricks have a significantly higher correlation coefficient value than hand-made bricks. Namely, the coefficient of correlation of R^2^ = 0.28 for the equation y = 135.88x − 30.60 was achieved for hand-made bricks, while machine-made bricks achieved the coefficient of correlation of R^2^ = 0.60 for the equation y = 710.00x − 491.80. A higher correlation coefficient value for machine-made bricks could be due to greater uniformity characteristics of those bricks compared to hand-made bricks. If such a correlation between the Maage factor and the ratio of compressive strengths before and after the freeze–thaw cycles for machine-made bricks is confirmed on a larger number of testing results, this parameter could be used as an additional method for assessing brick resistance to freezing and thawing cycles. This interesting observation will be further explored by the authors.

## 5. Conclusions

By analyzing the research results, the following conclusions could be made:

- Minor differences between the characteristics of raw materials had an important effect on the total share of pores in the case of the unfired brick specimens. It is not possible to conclude whether the raw material characteristics had an unambiguous influence on the compressive strength value after freeze–thaw cycles of the clay bricks;
- The mmethod of brick shaping influences the structure of pores of the unfired bricks; the machine-made bricks develop a greater share of large and a smaller share of medium pores than the hand-made ones. However, due to the transformation of the pore structure during the firing process, it is not possible to claim that the method of brick forming directly influenced their resistance to freeze–thaw cycles;
- A more extended period of brick retention at the highest achieved temperature results in a better resistance of the bricks to freeze–thaw cycles;
- All bricks proved to be resistant to freeze–thaw cycles according to the HRN B.D8.011 standard. The ratio of their compressive strengths before and after the exposure to freeze–thaw cycles was introduced as a quantitative parameter. For all the tested bricks, it was higher than 0.72 while the Maage factor of these bricks was in the range 27 to 100;
- A good relation between the Maage factor and the ratio of compressive strength before and after freeze–thaw cycles was observed in the case of the machine-made bricks. If such a correlation is confirmed on a larger number of testing bricks, the ratio of compressive strength before and after freeze–thaw cycles could be used as a new additional method for assessing brick resistance to freezing and thawing cycles.

## Figures and Tables

**Table 1:** Average values of the constituent elements in the samples of raw materials.

Chemical Element	Sample Label			
	S1		S2	
	Net	Conc. (%)	Net	Conc. (%)
Mg	67	1.696	139	3.530
Al	2410	9.118	2512	9.506
Si	14,490	32.030	14,968	33.086
P	–	–	52	0.047
K	4898	1.966	4385	1.760
Ca	2331	0.604	9404	2.438
Ti	3995	0.505	3886	0.491
Cr	194	0.011	195	0.011
Mn	1219	0.024	1613	0.032
Fe	109,117	5.569	105,983	5.409
Ni	–	–	195	0.005
Zn	656	0.016	647	0.015
Rb	557	–	739	–
Sr	514	0.015	693	0.021
Zr	1094	–	986	–

**Table 2:** Chemical composition of two raw materials expressed in oxide form.

Chemical Compound	Sample Label	
	S1, mass (%)	S2, mass (%)
SiO_2_	58.72	48.46
CO_2_	5.39	18.90
Al_2_O_3_	21.00	17.59
FeO	6.38	5.52
CaO	1.56	3.24
K_2_O	3.08	2.50
MgO	2.56	2.47
TiO_2_	0.75	0.72
Na_2_O	0.55	0.60

**Table 3:** Results of combustible, organic substances and carbonate content in the raw materials.

Sample Label	Combustible Substances (%)	Organic Substances (%)	Carbonates (%)
S1	4.3	2.2	1.3
S2	6.8	2.4	5.8

**Table 4:** Particle-size distribution of two raw materials.

Sample Label	Particle Description (Shape, Hard.)	Grain (mm)	G (%)	S (%)	M (%)	C (%)
•S1	round, hard and solid	1	000	4.77	52.74	42.49
°S2	round, hard and solid	1	0.00	4.66	53.67	41.67

Legend: G—gravel, S—sand, M—mold, C—clay.

**Table 5:** Used raw materials, way of shaping, maximal firing temperature and retention time at the maximal temperature.

Way of Shaping		Hand-Made (H)		Machine-Made (M)	
Raw material		S1	S2	S1	S2
Unfired specimens		S1H	S2H	S1 M	S2 M
Fired specimens	Thermal treatment	max T. 1030 °C	max T. 1060 °C	max T. 1030 °C	max T. 1060 °C
	Retention time 0.5 h	S1H1030-0.5h	S2H1060-0.5h	S1M1030-0.5h	S2M1060-0.5h
	Retention time 1.5 h	S1H1030-1.5h	S2H1060-1.5h	S1M1030-1.5h	S2M1060-1.5h

**Table 6:** Results of testing–assessment of brick resistance according to both direct and indirect methods, total volume of pores and Maage factor.

Specimen Label/ Tested Property	S1H1030-1.5h	S1H1030-0.5h	S2H1060-1.5h	S2H1060-0.5h	S1M1030-1.5h	S1M1030-0.5h	S2M1060-1.5h	S2M1060-0.5h
Resistance according to HRN B.D8.011	resistant	resistant	resistant	resistant	resistant	resistant	resistant	resistant
The ratio of compressive strengths	0.73	0.72	0.86	0.85	0.81	0.79	0.77	0.75
Total volume of pores, PV (cm^3^/g)	0.0961	0.1131	0.1051	0.1093	0.0891	0.1075	0.0964	0.1371
Maage factor (F_C_)	83	55	100	69	77	68	76	27
